# Occurrence and Health Risk Assessment of Aflatoxin B_1_ and Total Aflatoxins in Maize Flour From Jiroft City, Iran

**DOI:** 10.1002/fsn3.72246

**Published:** 2026-08-10

**Authors:** Zohreh Mashak, Ali Heshmati, Amir Sasan Mozaffari Nejad, Ali Asghar Kheirkhah Vakilabad, Saeme Asgari, Mohammad Mahdi Soori, Ali Habibi, Kasim Sakran Abass

**Affiliations:** ^1^ Department of Food Hygiene and Quality Control, Ka.C Islamic Azad University Karaj Iran; ^2^ Department of Food Science and Technology, National Nutrition and Food Technology Research Institute, Faculty of Nutrition Sciences and Food Technology Shahid Beheshti University of Medical Sciences Tehran Iran; ^3^ Bio Environmental Health Hazards Research Center Jiroft University of Medical Sciences Jiroft Iran; ^4^ Infectious Disease and Tropical Medicine Research Center Isfahan University of Medical Sciences Isfahan Iran; ^5^ School of Medicine Jiroft University of Medical Sciences Jiroft Iran; ^6^ Department of Biochemistry and Biophysics, TeMS.C Islamic Azad University Tehran Iran; ^7^ Department of Environmental Health Engineering, School of Public Health Jiroft University of Medical Sciences Jiroft Iran; ^8^ Department of Accounting and Management, Par.C Islamic Azad University Pardis Iran; ^9^ Department of Physiology, Biochemistry, and Pharmacology, College of Veterinary Medicine University of Kirkuk Kirkuk Iraq

**Keywords:** aflatoxin B_1_, dietary exposure, ELISA, maize flour, risk assessment, total aflatoxins

## Abstract

Aflatoxins are highly toxic secondary metabolites produced by certain species of *Aspergillus* that can contaminate food products and pose significant health risks to humans and animals. This study aimed to determine the occurrence and contamination levels of total aflatoxins (total AF) and aflatoxin B_1_ (AFB_1_) in maize flour marketed in Jiroft City, Kerman Province, Iran, and to assess the associated dietary exposure and health risk. Forty commercially packaged maize flour samples were collected from various local markets and supermarkets during June and July 2023. The concentrations of total AF and AFB_1_ were quantified using competitive enzyme‐linked immunosorbent assay (ELISA). All analyses were performed in duplicate to ensure reproducibility and accuracy. Detectable concentrations above the assay limit of detection (LOD) were found in 27 (67.5%) samples for AFB_1_ and 28 (70%) samples for total AF. Notably, AFB_1_ levels exceeded the European Commission (EC) permissible limit of 2 μg/kg in 14 samples (35%), while total AF levels were above the EC limit of 4 μg/kg in 8 samples (20%). Furthermore, 4 samples (10%) surpassed the regulatory threshold of 5 μg/kg for AFB_1_, while none exceeded the allowable limit of 30 μg/kg for total AF, as established by the Iran National Standards Organization (INSO). The estimated daily intake (EDI) of AFB_1_, calculated based on maize flour consumption and average body‐weight data for the adult population, was 2.29 × 10^−4^ μg/kg body weight/day. The calculated margin of exposure (MOE) was 1748.25, which is below the reference value of 10,000, indicating a potential health concern for consumers. Overall, the findings indicate that aflatoxin contamination in maize flour marketed in Jiroft remains a food safety concern and highlight the need for routine monitoring and strengthened quality control measures to minimize consumer exposure.

## Graphical abstract

Aflatoxin B_1_ and total aflatoxins were assessed in 40 commercial maize flour samples collected from Jiroft City, Iran. AFB_1_ and total aflatoxins were detected in 67.5% and 70% of samples, respectively. A total of 35% of samples exceeded the EU limit for AFB_1_, while 20% exceeded the EU limit for total aflatoxins.

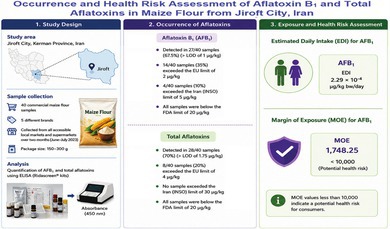

## 1 Introduction

Cereals, also known as grains, play a crucial role in the diets of all age groups worldwide, providing essential energy and nutrients necessary for growth and development, particularly in infants (Jeelani et al. 2020; Poutanen et al. 2022). Among these, maize ( *Zea mays* L.) is a staple food crop widely consumed globally, including in African countries, Latin America and some in Asia countries such as Iran (Maqbool and Beshir 2019). Maize and its derivatives, including maize flour, are prone to fungal contamination at various stages, including pre‐harvest, harvest, and post‐harvest processes (Sadik et al. 2023). This contamination can arise from various factors, including physical conditions (e.g., pH, light, moisture, temperature, water activity, relative humidity, and atmospheric gases), biological factors (e.g., fungal species, weeds, and insect damage), and nutritional factors (e.g., carbon, amino acids, nitrogen, lipids, and trace elements) (Kumar et al. 2021).

Fungal genera, including *Fusarium*, *Aspergillus*, *Penicillium*, *Claviceps*, and *Alternaria* can contaminate maize, leading to the production of secondary metabolites called mycotoxins, which may cause both acute and/or chronic toxicity in humans and animals (Heshmati et al. 2023; Kamkar, Yazdankhah, et al. 2014; Kamkar, Fallah, et al. 2014; Nejad et al. 2013; Rahimzadeh Barzoki et al. 2023). The exposure to mycotoxins is a global concern, exacerbated by the globalization of food trade (Anfossi et al. 2016). Of the 400 distinct mycotoxins that have been characterized, nearly 200 fungal species are responsible for their production (Lilburn 2025; Mozaffari Nejad and Giri 2015). Among the well‐known groups of mycotoxins are aflatoxins (AFs), fumonisins, ochratoxins, trichothecenes (e.g., deoxynivalenol, T‐2 toxin, and nivalenol), ergot alkaloids, patulin, and zearalenone (Awuchi et al. 2022; El‐Sayed et al. 2022; Heshmati et al. 2023; Khan et al. 2024). Ochratoxin A, fumonisins, and aflatoxins are especially detrimental to mammalian species, with aflatoxins being the most extensively studied mycotoxins worldwide (Awuchi et al. 2021, 2022; Bryden 2007; Khan et al. 2024). Currently, more than 5 billion people worldwide are exposed to varying levels of aflatoxins, often in an uncontrolled manner (Kensler et al. 2011).

Consumption of food contaminated with aflatoxins presents substantial public health risks due to their mutagenic, immunotoxic, carcinogenic, teratogenic, and nephrotoxic adverse effects (Heshmati et al. 2023; Patial et al. 2018). Approximately 18 types of aflatoxins have been identified; however, the most common and significant ones are aflatoxin B_1_ (AFB_1_), B_2_ (AFB_2_), G_1_ (AFG_1_), G_2_ (AFG_2_), M_1_ (AFM_1_), and M_2_ (AFM_2_) (Vijayasamundeeswari et al. 2010; Wacoo et al. 2014). AFB_1_, AFB_2_, AFG_1_, and AFG_2_ are frequently detected in human food, livestock feed crops, and agricultural commodities, whereas AFM_1_ and AFM_2_—derivatives of AFB_1_—are predominantly present in animal‐derived products, such as milk and dairy items (Mozaffari Nejad 2011; Mozaffari Nejad et al. 2020). The International Agency for Research on Cancer (IARC) has designated AFB_1_ as a Group I carcinogen, recognizing its potential to induce cancer in humans (Eslami et al. 2015; Mozaffari Nejad et al. 2014).

Given their toxic nature, the maximum allowable levels of aflatoxins in food must be rigorously monitored and regulated in compliance with national legislation. Although aflatoxin regulations have been established in more than 100 countries, there is a significant absence of international harmonization in these regulatory frameworks (Van Egmond and Jonker 2004; Heshmati and Mozaffari Nejad 2015; Heshmati et al. 2020; Mousavi Khaneghah et al. 2023). Globally, aflatoxin regulations vary significantly: INSO permits 30 μg/kg for total aflatoxins (total AF) and 5 μg/kg for AFB_1_ in cereals, the Food and Drug Administration (FDA) allows 20 μg/kg, and the European Commission (EC) enforces stricter limits of 4 μg/kg for total AF and 2 μg/kg for AFB_1_ (Akullo et al. 2023; European Commission (EC) 2023; Institute of Standards and Industrial Research of Iran 2020).

Research on mycotoxin contamination in agricultural and dairy products in Iran has intensified following the detection of aflatoxins and aflatoxigenic molds in various food items. Significant findings include the presence of aflatoxins in pistachios (Bagheri Marzooni et al. 2024), dried fruits (Heshmati and Mozaffari Nejad 2015; Heshmati et al. 2023), rice (Eslami et al. 2015), wheat (Heshmati et al. 2023; Rahimi et al. 2023), spices (Mozaffari Nejad et al. 2014; Nejad et al. 2013), cheese (Mozaffari Nejad et al. 2020; Tavakoli et al. 2012), yogurt (Heshmati et al. 2020; Tavakoli et al. 2013), raw milk, ultra‐high temperature (UHT), and pasteurized milk (Kamkar, Yazdankhah, et al. 2014; Kamkar, Fallah, et al. 2014; Mashak et al. 2016; Nejad et al. 2019; Rahimzadeh Barzoki et al. 2023). However, studies on maize (Karami‐Osboo et al. 2012) and maize flour (Rahimi et al. 2023) remain insufficient to determine the extent of contamination.

Aflatoxins in food commodities have been quantified and detected through various analytical techniques, including thin‐layer chromatography (TLC), high‐performance liquid chromatography (HPLC), and enzyme‐linked immunoassays (ELISA). While ELISA is favored for its simplicity, it is limited by lengthy incubation periods and multiple washing and mixing steps. Despite these drawbacks, ELISA remains extensively used in Iran due to its user‐friendly design, rapid processing, and capability for automation (Eslami et al. 2015; Heshmati et al. 2020; Kamkar, Yazdankhah, et al. 2014; Kamkar, Fallah, et al. 2014; Mozaffari Nejad et al. 2014; Nejad et al. 2013; Nejad et al. 2019).

The primary objectives of this study are: (1) to quantify the levels of AFB_1_ and total AF in maize flour by ELISA; (2) to evaluate these levels against the upper permissible limits set by EC and INSO; (3) to assess the risk of consumer exposure to aflatoxins in maize flour within Iran.

## 2 Materials and Methods

### 2.1 Materials

Aflatoxin ELISA kits were procured from R‐Biopharm Company (Darmstadt, Germany). Methanol (CH_3_OH) of analytical grade was obtained from Merck (Darmstadt, Germany).

### 2.2 Sample Collection

**FIGURE 1:**
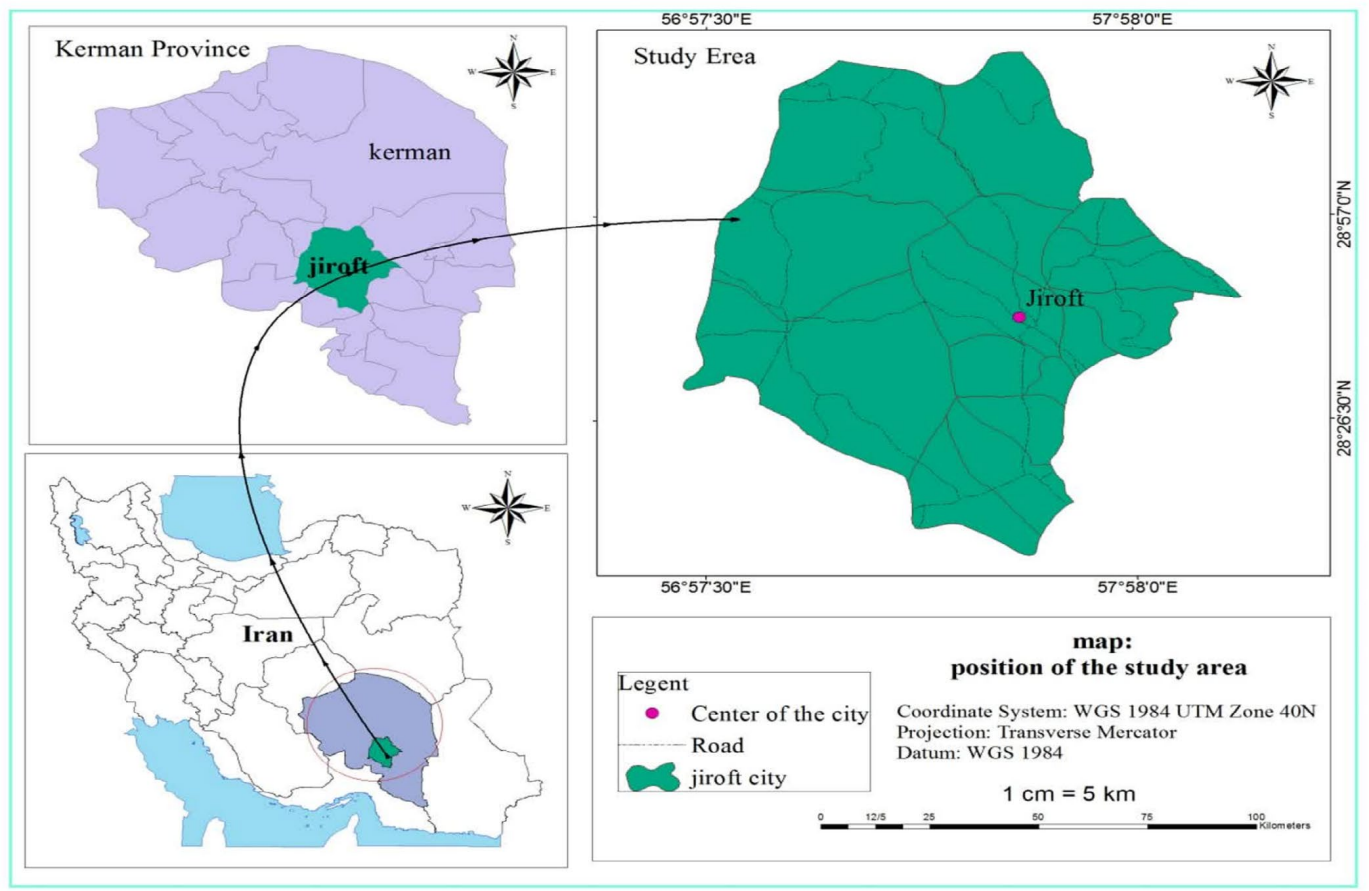
Location of the study area of Jiroft city, Kerman province, Iran.

Forty maize flour samples were collected by trained personnel from all accessible local markets and supermarkets in the Jiroft region, southeastern Kerman Province, Iran, during the summer months of June and July 2023. Jiroft, a major agricultural and commercial hub in southeastern Iran, serves as a representative and high‐risk region for investigating the safety of cereal‐based products (Figure 1). The selection of this location is justified by its hot and humid summer climate, which promotes fungal growth and increases the risk of mycotoxin contamination in stored food products (Mosazade et al. 2022). At each visited outlet, samples were collected for every unique commercial brand and package size (SKUs) available. Each sample consisted of 150–300 g of maize flour.

### 2.3 Sample Handling and Storage

The collected maize flour samples were immediately placed in polyethylene bags and labeled with a unique identification code, collection date, and sampling time. Upon arrival at the laboratory, the samples were visually inspected for any signs of fungal contamination. Subsequently, the samples were transferred to airtight plastic containers and stored at 4°C in a dark and dry environment to minimize fungal growth and preserve sample integrity. The sample handling and refrigerated storage conditions applied in the present study were consistent with procedures reported in previous investigations on aflatoxin contamination in cereal‐based products (Taheri et al. 2012; Jahanbakhsh et al. 2021; Heshmati et al. 2023). In the present study, all samples were analyzed within 14 days of collection for aflatoxin quantification at the Department of Chemistry, Islamic Azad University.

### 2.4 Sample Preparation and Aflatoxins Analysis by ELISA

Given the homogeneous nature of the maize flour samples, no specific sample preparation procedures were applied prior to the aflatoxins analysis.

Aflatoxin B_1_ (AFB_1_) and Aflatoxin Total were quantified using competitive enzyme‐linked immunosorbent assay (ELISA) kits (Ridascreen, R‐Biopharm AG, Darmstadt, Germany) according to the manufacturer's instructions.

For Aflatoxin Total determination, 2 g of maize flour was extracted with 10 mL of 70% (*v*/*v*) methanol–water and mixed using a vortex mixer for 10 min at room temperature. The suspension was centrifuged at 3500 rpm for 10 min and the supernatant was collected for analysis. Also, for AFB_1_ determination, 5 g of maize flour was mixed with 25 mL of 70% methanol and vortexed for 3 min. The extract was filtered through Whatman No. 1 filter paper, and 1 mL of the filtrate was diluted with 1 mL of distilled water prior to analysis.

The quantification of aflatoxins was performed using Ridascreen Aflatoxin Total Art. No. R4701 (R‐Biopharm AG 2016) and Ridascreen AFB_1_ 30/15 Art. No. R1211 kits. 50 μL of each standard solution and prepared sample extract were dispensed in duplicate into antibody‐coated microplate wells using fresh pipette tips for each transfer. Subsequently, 50 μL of enzyme conjugate and 50 μL of anti‐aflatoxin antibody solution were added to each well. The plate was incubated for 30 min at room temperature (20°C–25°C) in the dark. After incubation, the contents of the wells were discarded and each well was washed twice with 250 μL washing buffer (10 mM phosphate‐buffered saline containing 0.05% Tween 20, pH 7.4). Subsequently, 100 μL of substrate/chromogen solution was added to each well and the plate was incubated for 15 min at room temperature in the dark. The enzymatic reaction was stopped by adding 100 μL of stop solution (1 N H_2_SO_4_). Absorbance was measured at 450 nm using an ELISA microplate reader (ELx800, BioTek Instruments, USA).

A six‐point calibration curve was prepared for each assay. For total aflatoxins, standard concentrations were 0, 0.5, 1.5, 4.5, 13.5, and 40.5 μg/kg. For AFB_1_, standard concentrations were 0, 1, 5, 10, 20, and 50 μg/kg. Aflatoxin concentrations in the samples were calculated by interpolation from the corresponding calibration curves. All calibration standards and sample extracts were analyzed in duplicate, and the mean values were used for subsequent calculations.

According to the manufacturer's instructions provided with the Ridascreen ELISA kits used in this study, the limit of detection (LOD) was 1.75 μg/kg for total aflatoxins and 1 μg/kg for AFB_1_. These values represent the manufacturer's technical specifications and were not independently validated under the laboratory conditions of the present study. All analyses were performed in accordance with the manufacturer's protocol.

### 2.5 Risk Assessment of AFB_1_ Intake Through Maize Flour

For the assessment of AFB_1_ risk associated with maize flour consumption, the estimated daily intake (EDI) of this mycotoxin was calculated using Equation (1), as indicated below (EFSA 2010):

(1) $$\mathrm{EDI}\;\left(\mathrm{\mu g}/\mathrm{kg}\;\mathrm{BW}/\mathrm{day}\right)=\frac{\text{Daily consumption of maize flour}\;\left(0.0088\;\mathrm{kg}/\mathrm{day}\right)\times \text{Average}\;\mathrm{AFB}1\;\text{concentration}\;\left(1.82\;\mathrm{\mu g}/\mathrm{kg}\right)}{\text{Average body weight of Iranian adults}\;\left(70\;\mathrm{kg}\right)}$$

The daily consumption of maize flour was approximately 8.8 g/day (0.0088 kg/day), and the average body weight (BW) for Iranian adults was considered to be 70 kg (INSO 2021).

In addition, the margin of exposure (MOE) of AFB_1_ was calculated to evaluate its carcinogenic and genotoxic effect using Equation (2):

(2) $$\mathrm{MOE}=\mathrm{BMD}L_{10}/\mathrm{EDI}$$

According to the European Food Safety Authority (EFSA) guidelines, the benchmark dose lower confidence limit (BMDL_10_), corresponding to a 10% increase in the incidence of cancer (0.4 μg/kg bw per day), serves as the preferred reference point for MOE calculations (EFSA 2010).

An MOE value less than 10,000, particularly when derived from animal studies, is typically interpreted as a significant public health concern, necessitating subsequent risk management strategies (EFSA 2010).

### 2.6 Statistical Analysis

Statistical analysis was performed using SPSS software version 20 (IBM, PASW Statistics, USA). AFB_1_ and total AF concentrations were analyzed using descriptive statistics and are presented as mean ± standard deviation (SD). The frequency and percentage of samples with detectable aflatoxin concentrations above the LOD, the distribution of contamination levels, and the proportion of samples exceeding the maximum limits established by the INSO, EC, and FDA were calculated and reported. Because this was a descriptive study and no comparisons between independent groups were performed, tests of normality and inferential statistical analyses, including parametric and non‐parametric tests, were not applicable.

## 3 Results

The study was carried out for the analysis of aflatoxin B_1_ and total aflatoxin in 40 samples of maize flour in Jiroft, Iran. The incidence and occurrence levels of AFB_1_ and total AF in samples are presented in Tables 1 and 2, respectively. AFB_1_ was detected above the assay limit of detection (LOD; 1 μg/kg) in 27 samples (67.5%), whereas total AF was detected above the assay LOD (1.75 μg/kg) in 28 samples (70%). The mean concentrations of AFB_1_ and total AF were 1.82 ± 0.32 and 2.85 ± 0.52 μg/kg, respectively.

**TABLE 1:** Occurrence of aflatoxin B_1_ in maize flour in Jiroft, Iran.

Sample tested *N*	Mean ± SD^a^ (μg/kg)	Positive sample *n* (%)	Distribution of samples *n* (μg/kg)				Exceed legal limit *n* (%)	
			< LOD (1)^b^	1–2	> 2–5	> 5	INSO^c^	EC^d^
40	1.82 ± 0.32	27 (67.5)	13 (32.5)	13 (32.5)	10 (25)	4 (10)	4 (10)	14 (35)

^a^ Standard deviation.

^b^ The levels below the LOD.

^c^ Iran National Standards Organization (INSO) limit for AFB_1_ in maize is 5 (μg/kg).

^d^ European Commission (EC) limit for AFB_1_ in maize is 2 (μg/kg).

**TABLE 2:** Occurrence of total aflatoxins in maize flour in Jiroft, Iran.

Sample tested *N*	Mean ± SD^a^ (μg/kg)	Positive sample *n* (%)	Distribution of samples *n* (μg/kg)				Exceed legal limit *n* (%)		
			< LOD (1.75)^b^	1.75–4	4–30	> 30	INSO^c^	EC^d^	FDA^e^
40	2.85 ± 0.52	28 (70)	12 (30)	20 (50)	8 (20)	0	0	8 (20)	0

^a^ Standard deviation.

^b^ The levels below the LOD.

^c^ INSO limit for total aflatoxins in maize is 30 (μg/kg).

^d^ EC limit for total aflatoxins in maize is 4 (μg/kg).

^e^ FDA limit for total aflatoxins in maize is 20 (μg/kg).

Furthermore, 14 (35%) and 8 (20%) samples are found to be above the permissible limits for AFB_1_ and total AF as established by EC, that is, 2 μg/kg and 4 μg/kg, respectively. In addition, AFB_1_ concentrations in 4 samples (10% of all samples) exceeded the maximum allowable limit of 5 μg/kg set by the INSO. However, none of the analyzed samples surpassed the INSO maximum limit of 30 μg/kg for total AF. Furthermore, aflatoxin concentrations in all maize flour samples remained below the Food and Drug Administration (FDA) permissible limit of 20 μg/kg. For AFB_1_ intake through maize flour, the estimated daily intake (EDI) was 2.29 × 10^−4^ μg/kg BW/day, and the corresponding Margin of Exposure (MOE) was calculated as 1748.25. Since the MOE value of 1748.25 falls below the accepted safety limit of 10,000, these findings suggest that AFB_1_ intake from maize flour could pose a high health risk to consumers.

## 4 Discussion

Maize is one of the most widely produced cereal crops such as wheat and rice globally and highly susceptible to fungal contamination, particularly by *Fusarium* and *Aspergillus* species capable of producing aflatoxins (Afzaal et al. 2023; Visagie et al. 2024). Aflatoxins contamination may occur pre‐harvest or during harvesting, transportation, storage, and processing under favorable environmental conditions (Gómez‐Salazar et al. 2023; Mozaffari Nejad et al. 2014; Rahimzadeh Barzoki et al. 2023). In this study, the distribution of AFB_1_ and total aflatoxins was evaluated across positive and negative samples (Tables 1 and 2), and the results were compared with findings from previous research conducted in Iran and other countries (Table 3).

**TABLE 3:** Occurrence and levels of aflatoxin B_1_ and total aflatoxins in maize flour samples published in previous studies.

Location	No. of samples	Positive *n* (%)^a^	Detection method	Range (μg/kg)	References
Turkey	18	17 (94.4)	HPLC	0.13–12.87^b^	Algül and Kara (2014)
Pakistan	20	20 (100)	HPLC	2.0–34.0^b^	Firdous et al. (2014)
Turkey	24	16 (66.7)	HPLC	0.04–1.12^c^	Kara et al. (2015)
Turkey	69	11 (16%)	HPLC	0.28–22.65^b^	Şengül et al. (2016)
				0.37–24.54^c^	
Serbian	56	27 (48.2)	HPLC	< LOD—8.80^b^	Torović (2018)
				< LOD—9.14^c^	
Iran	40	25 (62.5)	LC/MS–MS	< LOQ—1060^b^	Amirahmadi et al. (2018)
Italy	8	NR	ELISA	5.0–7.6^c^	Sacco et al. (2020)
Uganda	29	17 (58.6)	ELISA	1.3–33.7^c^	Akullo et al. (2023)
Pakistan	80	21 (26.25)	TLC	1.51–38.54^b^	Naheed et al. (2024)

^a^ Percent (%): All percentages reported are based on the entire sample set.

^b^ Aflatoxin B1.

^c^ Total aflatoxins.

In a recent study from Uganda using the ELISA technique to determine total aflatoxin, Murokore et al. (2023) reported that 74.2% (23/31) maize flour samples were contaminated with total AF, and 18 (58.1%) exceeded the East African limit of 10 μg/kg. In comparison, the present study found contamination in 28 (70%) samples, although none exceeded the Iranian maximum limit of 30 μg/kg for total aflatoxin. Similarly, a study conducted in Kenya showed that all 27 maize flour samples collected from Nairobi, Eastern and Western regions contained detectable aflatoxin levels as determined by ELISA, but all were within the statutory limit of 10 μg/kg (Nduti et al. 2017). This consistent with our findings regarding the widespread presence of aflatoxins, although the concentrations in our samples were generally higher. In Tanzania, Kibwana et al. (2023) reported that 72% (36/50) of maize flour samples were contaminated with aflatoxins, as determined by ELISA, with concentrations ranging from 1.5 to 60.1 μg/kg; 28% of the samples exceeded the national limit of 10 μg/kg set by the Tanzania Bureau of Standards (TBS). In contrast, in the present study, only four samples (10% of all samples) exceeded the INSO limit of 5 μg/kg for AFB_1_, and no sample surpassed the INSO limit of 30 μg/kg for total aflatoxins.

Several studies in African countries have reported high average levels of aflatoxin contamination in maize flour. Notably, concentrations of AFB_1_ and total aflatoxins vary considerably across districts, likely due to geographical differences (Martinho et al. 2024). Moreover, differences between studies may also be influenced by the effects of climate change on fungal growth (Şengül et al. 2016). In Mozambique, Martinho et al. (2024) used HPLC and detected total aflatoxins in 40% (12/30) of maize flour samples, with concentrations ranging from 0.55 to 1.05 μg/kg and a mean AFB_1_ level of 0.31 ± 0.03 μg/kg. These levels are markedly lower than those observed in our study, where AFB_1_ was detected in all samples with a mean concentration of 1.82 ± 0.32 μg/kg.

In the current study, 14 (35%) and 8 (10%) of the maize flour samples exceeded the EC permissible limits for AFB_1_ and total aflatoxins, respectively. These findings show a relatively lower exceedance rate compared to a study conducted in Côte d'Ivoire, where Manizan et al. (2018) reported that all 18 (100%) maize flour samples analyzed without potash surpassed the EC regulatory limits for both AFB_1_ and total aflatoxins. In contrast, comparable evidence of aflatoxin contamination has been documented in Pakistan, where Alim et al. (2018) found that 50% (9/18) of maize flour samples were contaminated with aflatoxins, with 22% and 16% of samples exceeding the EC limits for AFB_1_ and total AF, respectively. Although the occurrence of contamination reported by Alim et al. (2018) aligns with our findings, the aflatoxin levels detected in the present study were generally higher, with the majority of samples exceeding the permissible limits. These discrepancies across studies may be explained by variations in climatic conditions, post‐harvest handling, storage practices, and differences in the commercial supply chain, particularly in warm regions such as Jiroft where environmental conditions are favorable for fungal growth and aflatoxin production. In Iran, Rahimi et al. (2023) using ELISA reported higher AFB_1_/AFs in maize flour than ours: AFB_1_ was present in all samples, with 80% and 35% exceeding the EC (2 μg/kg) and INSO (5 μg/kg) limits, respectively. By comparison, in our study 35% exceeded the EC limit and 10% exceeded the INSO 5 μg/kg threshold.

The present study assessed the potential health risk associated with AFB_1_ intake from maize flour consumed by the Iranian population. Our analysis yielded an estimated daily intake (EDI) of 2.29 × 10^−4^ μg/kg BW/day, and a corresponding MOE of 1748.25. Given that MOE values below 10,000 are widely accepted as indicative of significant public health concern for genotoxic carcinogens such as AFB_1_, these findings imply a considerable health risk for Iranian consumers. To provide a broader context for our findings, a comparison with recent studies investigating AFB_1_ in maize flour from other regions is warranted. Studies in Sub‐Saharan Africa and Latin America frequently report high AFB_1_ exposure levels and lower MOE. For instance, Martinho et al. (2024) in maize flour produced in Mozambique reported alarmingly low MOE values of 231–243, concluding a potential risk of hepatocarcinoma. Furthermore, Sandoval et al. (2019) in Mexico found AFB_1_ in nixtamalized maize with EDI and MOE values ranging from 0.7 to 8.5 μg/kg Bw/day (0.0007–0.0085 μg/kg BW/day) and 20–257, respectively. These extremely low MOE values reported in Mozambique and Mexico underscore an acute risk profile in those regions, driven by factors such as high consumption and/or specific contamination patterns.

In addition, our calculated EDI (2.29 × 10^−4^ μg/kg BW/day) was considerably lower than the daily exposure levels reported for Tanzania (0.10–0.26 μg/kg/day) by Mfinanga Mariam et al. (2024). This variability in risk magnitude across regions is likely influenced by differences in contamination prevalence, average consumption rates, analytical methodologies, and food processing techniques. The collective evidence from MOE‐based assessments, including the current study and those from Mozambique and Mexico, points toward a pervasive and concerning threat regarding dietary AFB_1_ exposure from maize flour.

### 4.1 Study Limitations

The present study has several limitations that should be considered when interpreting the findings. First, although the sampling strategy was designed to maximize market coverage by including all accessible local markets and supermarkets, different commercial brands, production batches, and package sizes available during the study period, the relatively limited sample size and sampling from a single geographic location (Jiroft) may limit the generalizability of the findings to other regions of Iran. Second, sampling was conducted over a two‐month period and therefore did not account for possible seasonal variations in aflatoxin contamination. Third, aflatoxin concentrations were determined using a validated commercial ELISA kit, and confirmatory analysis by chromatographic techniques, such as HPLC or liquid chromatography–tandem mass spectrometry (LC–MS/MS), was not performed. In addition, laboratory‐specific analytical validation, including spike‐recovery experiments, repeatability, reproducibility, and matrix‐specific performance evaluation, was not performed, and the analytical performance characteristics reported in this study were based solely on the manufacturer's technical specifications. Finally, the dietary exposure assessment was based on available consumption data and default body‐weight assumptions, which may not fully reflect individual dietary habits and exposure variability. Despite these limitations, the study provides valuable baseline information on the occurrence of aflatoxins in maize flour marketed in Jiroft. Future multicenter studies involving larger sample sizes, seasonal sampling, confirmatory chromatographic analyses, and comprehensive laboratory‐specific method validation are recommended to provide a more robust assessment of aflatoxin occurrence, dietary exposure, and associated health risks.

## 5 Conclusion

This study demonstrates the presence of aflatoxin contamination in commercially available maize flour from Jiroft city in Iran. Although most samples complied with national regulatory limits, a number of samples exceeded international standards, indicating potential food safety concerns. The risk assessment findings underscore the importance of minimizing dietary exposure to AFB_1_ through enhanced control measures in maize flour production and storage. Implementing continuous surveillance and adopting enhanced post‐harvest and handling practices are essential steps toward reducing contamination levels and safeguarding public health.

## Author Contributions

**Zohreh Mashak:** investigation, visualization, writing – original draft, methodology, validation. **Amir Sasan Mozaffari Nejad:** conceptualization, investigation, funding acquisition, writing – original draft, writing – review and editing, visualization, project administration, supervision, resources, data curation, software, methodology. **Saeme Asgari:** conceptualization, investigation, writing – review and editing, validation, methodology, formal analysis, supervision. **Ali Asghar Kheirkhah Vakilabad:** writing – original draft, writing – review and editing, investigation. **Ali Heshmati:** conceptualization, investigation, validation, software, data curation, visualization. **Kasim Sakran Abass:** writing – review and editing, investigation, visualization. **Mohammad Mahdi Soori:** writing – review and editing, validation, software, methodology. **Ali Habibi:** writing – original draft, writing – review and editing, software.

## Ethics Statement

The authors have nothing to report.

## Consent

The authors have nothing to report.

## Conflicts of Interest

The authors declare no conflicts of interest.

## Data Availability

The data that support the findings of this study are available from the corresponding author upon reasonable request.
